# Inhibition of 19S proteasome-associated deubiquitinases by metal-containing compounds

**DOI:** 10.18632/oncoscience.167

**Published:** 2015-05-31

**Authors:** Ningning Liu, Hongbiao Huang, Q. Ping Dou, Jinbao Liu

**Affiliations:** ^1^ State Key Lab of Respiratory Disease, Protein Modification and Degradation Lab, Department of Pathophysiology, Guangzhou Medical University, Guangdong, China; ^2^ Guangzhou Research Institute of Cardiovascular Disease, The Second Affiliated Hospital of Guangzhou Medical University, Guangzhou, Guangdong, People's Republic of China; ^3^ The Molecular Therapeutics Program, Barbara Ann Karmanos Cancer Institute, and Departments of Oncology, Pharmacology and Pathology, School of Medicine, Wayne State University, Detroit, Michigan, USA

**Keywords:** 19S-associated deubiquitinases, copper complexes, gold complexes

## Abstract

Copper and gold complexes have clinical activity in several diseases including cancer. Recently, we have reported that the anti-cancer activity of copper (II) pyrithione CuPT and gold (I) complex auranofin is associated with targeting the 19S proteasome-associated deubiquitinases (DUBs), UCHL5 and USP14. Here we discuss metal DUB inhibitors in treating cancer and other diseases. (from Editor). Several copper and gold complexes have clinical activity in treating some human diseases including cancer. Recently, we have reported that the anti-cancer activity of copper (II) pyrithione CuPT and gold (I) complex auranofin is tightly associated with their ability to target and inhibit the 19S proteasome-associated deubiquitinases (DUBs), UCHL5 and USP14. In this article we review small molecule inhibitors of DUBs and 19S proteasome-associated DUBs. We then describe and discuss the ubique nature of CuPT and auranofin, which is inhibition of 19S proteasome-associated UCHL5 and USP14. We finally suggest the potential to develop novel, specific metal-based DUB inhibitors for treating cancer and other diseases

## INTRODUCTION

### Ubiquitin-proteasome system and copper/gold complexes

The ubiquitin-proteasome system (UPS) mediates protein degradation through a cascade process. The target protein can be bonded to ubiquitin (Ub) through Ub-activating enzymes (E1), Ub-conjugating enzymes (E2) and Ub-protein ligases (E3). The 26S proteasome, a multisubunit protein complex that exerts function with ATP, contains two 19S proteasomes in both sides and a cylinder-shaped 20S proteasome in the middle. Ubiquitylated protein is degraded by 20S proteasome which has three related active proteolytic sites, chymotrypsine-like, trypsine-like and caspase-like. One physiological role of the 19S proteasome is to unfold and remove the Ub chains from the target protein in order to send the protein to 20S core [1-3].

Since the proteasome inhibitor bortezomib (Velcade, PS341) has been approved by FDA as a potent anti-multiple myeloma drug, the proteasome-mediated degradation pathway has proven to be an important target for developing novel drugs for the treatment of cancer and other diseases. Our laboratory and others have reported that the chymotrypsine-like activity of the 20S proteasome is the important drug target for several medicinal and metal-containing antitumor proteasome inhibitors. Shikonin, a natural naphthoquinone isolated from the traditional Chinese medicine Zi Cao, inhibits the proteasome chymotrypsin-like activity *in vitro* and *in vivo*, associated with induction of the tumor cell death through accumulation of proapoptotic proteins IkB-α, Bax and p27 [4]. Sanggenon C, a natural prenylated flavonoid, inhibits tumor cellular proteasomal chymotrypsin-like activity and cell growth [5]. Gambogic acid, a natural compound derived from Chinese herbs approved by the Chinese Food and Drug Administration for clinical trials in cancer patients, produces tissue-specific proteasome chymotrypsin-like inhibition and tumor-specific toxicity [6] and overcomes imatinib resistance by inducing proteasome inhibition and caspase-dependent Bcr-Abl downregulation [7]. Several copper compounds such as NCI-109268 and bis-8-hydroxyquinoline copper(II) [Cu(8-OHQ)(2)] can inhibit the chymotrypsin-like activity of purified 20S proteasome and cellular proteasome activity [8]. Disulfiram, a member of the dithiocarbamate family capable of binding copper and an inhibitor of aldehyde dehydrogenase, inhibits proteasome chymotrypsin-like activity in copper-containing tumor cells and xenografts [9]. Clioquinol, a therapeutic agent for Alzheimer's disease, is able to target tumor proteasome *in vivo* in a copper-dependent manner, resulting in formation of an active AR inhibitor and apoptosis inducer that is responsible for its observed antiprostate tumor effect [10]. Gallium(III)-containing complexes show promising antineoplastic effects particularly in lymphomas and bladder cancer by acting as potent proteasome inhibitors [11]. The copper complexes binding with 1,10-phenanthroline as the third ligand also serve as potent, selective proteasome inhibitors and apoptosis inducers in human tumor cells, and these ternary complexes may be good potential antitumor drugs [12]. Some synthetic gold(III) dithiocarbamate complex shows the inhibitory activity to a purified 20S proteasome and 26S proteasome in intact highly metastatic breast cancer cells with the accumulation of ubiquitinated proteins and induction of apoptosis, which is also exhibiting inhibition in breast tumor-bearing nude mice [13-14]. Two gold(III)-dithiocarbamato peptidomimetics target the MDA-MB-231 (resistant to cisplatin) breast cancer cell cultures and xenografts though proteasome chymotrypsin-like activity inhibition [15].

From the above observations we believed that metal complexes act as the potential antitumor drugs *via* targeting proteasome activity. In fact, it has been known that the platinum-based drug cisplatin, one of the most effective chemotherapy agents, exerts the antitumor activity. Furthermore, metal complexes have already been used as the potential antitumor drugs for treatment of various human diseases for centuries [16-18].

Copper has a long history in medical application [19]. Copper in cells is proved to trigger the ubiquitin aggregation [20], and binds certain types of organic ligands to form potent proteasome inhibitors and induce apoptosis *in vitro* and in *vivo* [8]. The reduced (Cu+) or oxidized (Cu2+) state of copper drives its diverse roles in structure and catalysis. Since Cu+ has an affinity for thiol and thioether groups and Cu2+ exhibits a preferred coordination to oxygen or imidazole nitrogen groups, these metal ions can participate in a wide spectrum of interactions with proteins to exert diverse structures and biochemical reactions [21]. Phosphine Cu+ complex (CP) as an efficient *in vitro* antitumor agent could induce ER-stress-mediated apoptosis in colon cancer cells and primary cells from B-acute lymphoblastic leukemia patients, and sensitize B-acute lymphoblastic leukemia cells to chemotherapeutic agents, associated with inhibition of all three proteolytic enzyme activities of the 20S proteasome [22-23]. Cu2+ appears to induce fibril-fibril association without affecting fibrillar structure of Alzheimer's disease amyloid-beta peptide [24]. The thioxotriazole Cu2+ complex A0 exhibits a significantly higher cytotoxic activity in the human fibrosarcoma cells with non-apoptotic programmed cell death [25]. A0 also causes paraptotic cell death via eIF2α phosphorylation and unfolded protein response (UPR) in human cancer cells [26].

Gold also has a long history as a potent therapeutic agent [27-31]. Gold (I) compounds such as auranofin have been used clinically to treat rheumatoid arthritis for many years. However, auranofin also exerts immunosuppressive activity which may through inhibiting MHC-restricted antigen presentation in professional antigen-presenting cells [32], and exhibits potent antimalarial effects by causing severe intracellular oxidative stress in vitro [33]. Auranofin can inhibit thioredoxin reductase-1, serving as a promising approach for lung cancer therapy [34]. Auranofin induces ER-stress response in cultured and primary chronic lymphocytic leukemia cells [35]. Auranofin also increases levels of pro-apoptotic proteins Bax and Bim and reduces anti-apoptotic protein Bcl-2 in ovarian carcinoma cells, and activates caspase-3-mediated apoptosis in a FOXO3-dependent manner [36]. Gold(III) and organogold(III) compounds have been reported as potential antitumor agents [37-39]. Two gold(III) methylsarcosinedithiocarbamate derivatives, combining cytostatic and apoptotic activity with reduced nephrotoxicity for the management of myeloid leukemia, can down-regulate Bcl-2 and upregulate Bax to induce cell death [40]. Iminophosphorane-organogold(III) complexes can induce tumor cell death through mitochondrial ROS production [41].

### Deubiquitinases (DUBs) and their small molecules inhibitors

The UPS includes the large family of DUBs. DUBs mediate the deubiquitination of the proteolytic process of the UPS. DUBs belong to the superfamily of proteases. The human genome encodes at least 98 DUBs which belongs to 6 subfamilies based on sequence and structural similarity: ubiquitin carboxy-terminal hydrolases (UCHs), ubiquitin-specific proteases (USPs), ovarian-tumor proteases (OTUs), Machado-Joseph disease protein domain proteases, JAMM/MPN domain-associated metallopeptidases (JAMMs) and monocyte chemotactic protein-induced protein (MCPIP) family [42-44]. All these are cysteine proteases except JAMMs family, which belongs to the metalloproteases catalytic class.

A number of DUBs have been shown to play a role in the process of diseases. UCHL1 is associated with a rare form of Parkinsonism and its accumulation is likely to play a pathological role in inclusion formation in Parkinson's diseases [45]. Inhibition of USP1 leads to hyperaccumulation of monoubiquitinated FANCD2, a protein that appears to be critical in the repair of DNA damage [46]. High expression levels of USP28 are found in colon and breast carcinomas, associated with the stabilization of MYC in the nucleus for tumor cell proliferation [47]. USP2 stabilizes cyclin D1 in order to maintain human cancer cell growth; targeting USP2 is therefore an effective approach to induce growth suppression of cancer cells addicted to cyclin D1 expression [48]. USP9X can stabilize MCL-1 to promote tumor cell survival through removing the Lys 48-linked polyubiquitin chains that normally mark MCL-1 for proteasomal degradation, which serves as a prognostic and therapeutic target of human malignancies [49]. Cylindromatosis tumor suppressor gene (CYLD), a member of the USPs family, was first identified as a tumor suppressor gene in the regulation of NF-κB activation [50]. A20, a member of the OTUs family, was first discovered after TNF-alpha treatment from mice deficient for A20 developed severe inflammation related with hypersensitivity to TNF-alpha [51].

Some DUB inhibitors have been reported to influence specific DUBs with diverse function. Ubiquitin aldehyde (Ubal) and ubiquitin vinylsulfone (UbVS) were used previously as irreversible DUB inhibitors mainly for the purpose of analysis of the three-dimensional structure of DUB enzymes, because their high molecular weight and limited specificity restrict their development to therapeutic drugs [52-53]. WP1130 is a small molecule that directly inhibits USP9X, USP5, USP14 and UCH37, all known as regulators of survival protein stability and 26S proteasome function; WP1130-mediated DUBs' inhibition down-regulates the antiapoptotic protein MCL-1 and upregulates the proapoptotic proteins p53 [54]. WP1130-induced unfolded protein response (UPR) blocks specific viral infection via activating the X-box binding protein-1 (XBP-1) in murine macrophages, suggesting its potential use for broad spectrum antiviral therapies [55]. P5091 is a small molecule-selective inhibitor of USP7, discovered by using a ubiquitin-phospholipase A_2_ enzyme reporter assay in a high throughput screening for inhibitors of USP7 from a diversity-based library of small molecules. Supported by *in vivo* and *in vitro* data, P5091 acts as a potential anti-multiple myeloma drug to overcome bortezomib resistance [56]. P22077, another USP7 inhibitor, stabilizes p53 by inducing HDM2 protein degradation in neuroblastoma (NB) cells and inhibits the xenograft growth of three cell lines in the NB mouse model [57]. PR-619 has been characterized as a broad-range, reversible DUB inhibitor of ubiquitin isopeptidases with potential to be developed into an anticancer chemotherapeutic agent. PR-619 also affects the microtubule network and causes protein aggregate formation in neural cells with implications in neurodegerative diseases [58,59].

### 19S proteasome-associated DUBs and their inhibitors

There are three important DUBs associated with the 19S proteasome, the JAMM family member POH1 (also known as RPN11/pda1/S13/mpr1), the USP family member USP14 and the UCHs family member UCHL5 (also known as UCH37). POH1 is a Zn-dependent metalloprotease, whereas USP14 and UCHL5 are cysteine proteases [60].

POH1 is responsible for substrate deubiquitination during protein degradation in proteasome [61]. Overexpression of POH1 in mammalian cells may help tumor cell escape from chemotherapeutic agents through increased cell growth and resistance to cytotoxic drugs [62]. POH1 contributes to the regulation of c-Jun ubiqutination, stability and subcellular localization, suggesting a novel mechanism of c-Jun regulation in mammalian cells [63]. As a required enzymatic subunit of the 19S proteasome, the JAMM motif of POH1 is essential for cell viability [64]. It appears that POH1 cleaves at the base of the ubiquitin chain where it is linked to the target protein, whereas USP14 and UCHL5 mediate a stepwise removal of ubiquitin from the protein by disassembling the chain from its distal tip [65]. The RNAi of UCHL5 or USP14 alone does not affect cell growth and proteasome composition but accelerates cellular protein degradation; however, RNAi of both UCHL5 and USP14 can inhibit cellular protein degradation. Thus proper proteasomal processing of ubiquitylated substrates needs POH1 plus either UCHL5 or USP14 [66].

USP14 regulates both the nature and magnitude of proteasome activity [67]. The role of USP14 in disease development remains unclear. In USP14-deficient ax(J) mice, the nervous systemic endogenous tau and ataxin-3 levels decrease and the phosphorylated tau levels increase which is accompanied by increased levels of activated phospho-Akt, phosphorylated MAPKs, and inactivated phospho-GSK3β, begging a better understanding of the treatment with chronic neurological diseases through the cellular pathways regulated by the proteasome [68]. The USP14 deficiency in the ax(J) mice contributes to diseases characterized by synaptic dysfunction [69]. Genetic and chemical suppression of USP14 activity caused an increase in Dishevelled (Dvl) polyubiquitination and significantly impaired downstream Wnt signaling, suggesting an oncogenic role for USP14 through Wnt/β-catenin signaling enhancement [70]. USP14 binding to the IRE1 protein for ER stress regulation indicates an important role in mutant Huntingtin-induced cell toxicity and the murine norovirus-caused infections [55, 71]. The USP14 expression is upregulated in non-small cell lung cancer (NSCLC) cells, especially in adenocarcinoma cells, suggesting its tumor-promoting function. Downregulation of USP14 expression is related to β-catenin degradation that blocks the NSCLC cell cycle progression [72]. Up-expression of miR-4782-3p is related with favorable prognosis in NSCLC cells through decreasing the USP14 expression [73]. Although USP14 usually plays an inhibitory role in protein degradation, overexpression of USP14 induced I-κB degradation by linking RelA, the binding partner of I-κB, leading to increased cytokine release in lung epithelial cells [74].

UCHL5 is responsible for the ubiquitin isopeptidase activity in the 19S proteasome regulatory complex, acting as the constituent subunit like the POH1 subunit. Rpn13 binds the carboxy-terminal tail of UCHL5 in order to recruit it into proteasome through binding to Rpn2 subunit of the 19S complex [75-77]. Rpn13 may involve the iNOS/IκB-α degradation and the interaction of iNOS/IκB-α with UCHL5, indicating the iNOS/IκB-α as the substrates for the Rpn13/UCHL5 [78]. UCHL5 interacts with Smads and reverses Smurf-mediated ubiqutination, and it can also deubiquitinate and stabilize the type I TGF-β receptor in cells [79]. UCHL5 protein expression is increased and required in high glucose-induced mouse mesangial cells in a possible PI3K-dependent fashion. UCHL5 shRNA attenuates high glucose-induced TGF-βR1 protein expression, TGF-βR1 protein deubiquitination, p21 (WAF1) fibronectin protein expression, and cell hypertrophy [80]. UCHL5 may play an important role in apoptotic death through altering Bax/Bcl-2 ratio and caspase-9/3 activities in A549 cells [81]. Overexpression of UCHL5 in hepatocellular carcinoma (HCC) cancerous cells can promote cell migration and invasion through interacting and deubiquitinating PRP19, an essential RNA splicing factor. UCHL5 expressing *in vitro* and *in vivo* suggests its predictor role for HCC recurrence after curative resection [82].

IU1 is a specific inhibitor of USP14 vs. other human DUBs including 19S proteasome DUBs, which is also able to enhance the proteasome activity [83]. This small-molecule inhibitor can prevent ventilator-induced lung injury in rats by attenuate intrapulmonary inflammatory response [84]. IU1 also inhibits Dengue virus replication, providing new targets for therapeutic intervention against viruses from multiple families [85]. Based on the structure of IU1, a group of tricyclic heterocyclics have been developed to specifically inhibit USP14 activity. These compounds could accelerate the degradation of abnormal and/or misfolded proteins in the cells by targeting USP14 [86].

Another small molecule b-AP15 is first reported as a new tumor cell inhibitor to 19S regulatory-particle-associated deubiquitinases USP14 and UCHL5 activity without affecting 20S core-particle-associated proteolytic activity [87]. b-AP15 triggers apoptosis in the multiple myeloma (MM) cells and patient MM cells through caspase activation and overcomes 20S proteasome inhibitor bortezomib resistance [88-89]. Exposure of tumor cell lines to b-AP15 resulted in increased TRAIL-R2 expression and enhanced sensitivity to TRAIL-mediated apoptosis and cell death *in vitro* and *in vivo* [90]. b-AP15-induced sensitization to TRAIL-mediated apoptosis could be used as a novel strategy to augment the anticancer effects of adoptively infused NK and T cells in patients with cancer [91]. The effect of b-AP15 in cells can be impaired by the antioxidant N-ethylmaleimide that could cause inhibition of selenoprotein thioredoxin reductase to trigger oxidative stress [92]. Azepan-4-ones, similar to b-AP15 also possess the inhibitory activity to USP14 and UCHL5, but this set of compounds do not affect non-proteasomal DUBs. They are described as effective compounds for the treatment of tumor resistance especially the bortezomib-refractory tumors caused by over-expressing Bcl-2 protein [93].

AC17, a 4-arylidene curcumin analog synthesized by Zhou et al., serves as an irreversible 19S-associated DUB inhibitor while it does not affect 20S proteasome proteolytic activities. However, the AC17 targeting sites in 19S-related DUBs have not been determined. The inhibition of 19S proteasome DUBs by AC17 suppresses cell proliferation by blocking NF-κB activation and increasing p53, MDM2 and p21 expression in both human lung adenocarcinoma A549 cells and xenograft model [94].

### CuPT and auranofin on 19S proteasome-associated UCHL5 and USP14

The application of the metal complex proteasome inhibitors in cancer therapy suggests that targeting the UPS by inhibiting the proteasome-associated DUBs may have potential uses clinically. Consistently, a reported panel of 2-phenylpyrindine gold(III) complexes containing a dithiocarbamate ligand display a promising inhibitory effect on UCHs and significant tumor cell cytotoxicity [95]. Two important recent reports on proteasome-associated metal complexes as DUBs inhibitors display promising *in vivo* anti-cancer activities against several cancers, further lending support to this view (Table 1) [96-97].

**Table 1:** Classification and summary of the 19S-associated DUB inhibitors

Inhibitor	19S-associatedDUB/s	Substrate/s	First-reportedFunction	Related cell lines and tumor model	References
WP1130	UCHL5, USP14 (and USP9X, USP5)	MCL.1, p53	Induces tumor cells apoptosis through selectively inhibiting DUBs	MM1.S, Z138, HEK293T	[54]
IU1	USP14	Tau, TDP-43, cODC-EGFP	Accelerates the degradation of oxidized proteins and enhances resistance to oxidative stress*in vitro*	Primary mouse embryonic (MEFs), HEK293, HeLa	[83]
Tricyclic heterocyclics (IU2-6)	USP14	Tau	Accelerate the degradation of abnormal and/or misfolded proteins in cells	MEF	[86]
b-AP15	UCHL5, USP14	p53, ODC-1, CDKN1B, CDKN1A, Caspase-3, PARP	Inhibits tumor progression *in vitro* and *in vivo*	HCT-116, SCID mice with FaDu human tumor xenografts, C57BL/6J mice with syngenic LLC tumor, BALB/C mice with orthotopic breast carcinomas (4T1), Xenograft-derived CK18 in circulation	[87]
Azepan-4-ones	UCHL5, USP14	Bcl-2	Inhibit the refractory tumor	Multiple myeloma and other solid tumor malignancies like lung, prostate, colon, ovary, pancreas, breast, neck and head cancers	[93]
AC17	19S-associatedDUBs	NF-κB, p53, MDM2, p21	Suppresses the cell proliferation.	A549, BALB/C nude mice with A549 xenograft.	[94]
AuIII	UCHL1-C UCHL3-C UCHL5-C(C: a cysteine active site)	Caspase-7, PARP	Induces cell-cycle arrest, apoptosis and anti-angiogenic property in breast cancer cells	MCF-7, MDA-MB-231, HeLa, MIHA	[95]
CuPT	UCHL5, USP14	p21, p27, Bax, IκB-α, PARP, Caspase-3, Caspase-9, Caspase-8,	Selectively inhibits tumor growth *in vivo* and induces cytotoxicity*in vitro* and *ex vivo*	MCF-7, HepG2, U266, NCI-H929, GFPu-HEK293, Primary acute myeloid leukemia cells (AML), BALB/C nude mice with HepG2/NCI-H929 xenografts	[96]
Auranofin	UCHL5, USP14	c-Jun, p21, IκB-α, NF-κB, CHOP, Caspase-3,8,9,12, PARP	Inhibits tumor growth *in vivo* and induces cytotoxicity*in vitro* and ex vivo	MCF-7, HepG2, GFPu-HEK293, Primary acute myeloid leukemia cells (AML), BAB/C nude mice with HepG2/MCF-7 xenografts	[97]

Copper pyrithione (CuPT) is an alternative to tributyltin for antifouling paint biocides [98]. We investigate the inhibitory effect of CuPT on USP14/UCHL5 activity and the relationship to cellular apoptosis *in vitro* and *in vivo*, towards the goal of developing novel DUB inhibitors and clinical anticancer strategy. We first compare the effect of PT/CuCl alone and 2:1 PT/CuCl combination on a number of tumor cell lines, including human breast MCF-7, human multiple myeloma U266 and NCI-H929, and human hepatoma SMMC-7721. The results show that the combination of PT and CuCl induces cytotoxicity much more effectively than PT or CuCl along. However, we found that the PT+CuCl, but not PT+H O induced the UPS inhibition. We also observed that the combination (CuPT) of 1 Cu molecule and 2 PTs induced cytotoxicity in multiple cancer cell lines (24h IC values: MCF7 0.375 μM, U266 0.130 μM, and HepG2 0.495 μM) and primary cancer cells from patients with acute myeloid leukemia (AML) (average 24h IC50=57.03 nM while CTR=101.08 nM). CuPT induced UPS malfunction which is similar to the mixture of PT and copper in human hepatoma HepG2 cells and GFPu-HEK293 cells with cellular ubiquitinated and GFP protein aggregation. It can only inhibit the chymotrypsine-like (but not caspase or trypsin-like activity) at high doses (>1 μM) of purified 20S proteasome under cell-free condition. Interestingly, low concentration of CuPT exerts inhibition on 19S-associated DUBs, USP14 and UCHL5. The computational study supports the docking affinity between CuPT and the 19S-associated DUBs and suggests its relationship with inhibiting USP14 and UCHL5 activities. CuPT (0.5 μM) can inhibit the DUB activity significantly on the purified 26S proteasome as the same as the pan DUB inhibitor NEM (with the concentration at 2 mM). Also, CuPT (0.5 and 1 μM) blocks 26S proteasome-cleaved K48-linked Ub chains and is able to compete with UbVS's binding with both USP14 and UCHL5 in a dose-dependent manner. Daily treatment of mice bearing HepG2 and NCI-H929 xenografts with 2.5 mg/kg/day CuPT for 15 and 5 days resulted in significant tumor growth inhibition. Associated with this growth inhibition, a significant accumulation of ubiquitinated proteins, K48-linked proteins, p21, p27, Bax and IκB-α are detected.

Auranofin, a gold-containing compound, is clinically used to cure rheumatic arthritis, and recently approved by US food and drug administration for phase II clinical trial to treat cancer. We report for the first time that this clinically used metal complex drug auranofin is a specific inhibitor of the 19S-associated DUBs, USP14 and UCHL5 with promising antitumor effects. We showed that auranofin potently inhibited proliferation of two tumor cell lines, HepG2 and MCF-7, with 24h IC50 values of 0.43 μM and 1.50 μM, 48h IC50 of 0.17 μM and 0.41 μM, respectively. Moreover, auranofin treatment for 24h induces apoptotic morphological changes in HepG2 (at 0.25 μM) and MCF-7 (0.5 μM) cells. The flow cytometry data also supports the results observed with fluorescence microscopy. Apoptotic specific changes in caspase and PARP proteins are showed in dose-dependent manner after auranofin treatment, demonstrating that the auranofin-triggered apoptosis is related with caspase activation. Next, we found that auranofin at 0.5 μM for 3h induces marked increases in total, K48-and K63-linked ubiquitinated proteins, indicating its UPS inhibitory effect. GFPu proteins and fluorescent images also supported the inhibitory effect of auranofin. The proteasome substrate proteins p21 and c-Jun increase after 9h treatment of auranofin. Importantly, we observed that the K48-linked accumulation induced by therapeutic dose of auranofin (0.5 μM) is similar to bortezomib at dose between 20 and 40 nM in K562 cells. These results indicate that a therapeutic dose of auranofin can achieve bortezomib's UPS inhibitory effect. The computational molecular docking, DUB activity, K48-linked Ub chains cleavage and HA-UbVS (HA-tagged ubiquitin-Vinyl Sulfone, which covalently binds to the active sites of the cysteine proteasome families of DUBs) competitive binding experiments are all implemented to prove auranofin's inhibitory activity to 19S-associated DUBs. The computational model also indicates that an active metabolite of auranofin can inhibit UCHL5 and USP14.

Ub-AMC is a fluorogenic substrate for a wide range of DUBs including UCHs and USPs. Using Ub-AMC as a DUB substrate, auranofin slightly inhibits the total cytoplasmic DUB activities while completely inhibits the purified 26S proteasome-associated DUB activities at 2 μM (as the same effect as 2 mM NEM). We used NAC, a thiol-containing compound, to block the active site of auranofin and found NAC recovers most auranofin-mediated DUB inhibition to purified 26S proteasome, which confirms the computational model results that auranofin targets proteasome-associated UCHL5 and USP14. K48-linked Ub chains cleavage is partially blocked by auranofin in a dose-dependent manner. HA-UbVS pretreatment of auranofin could bind the HA-tagged UbVS in the purified 26S proteasome, supporting that auranofin inhibits UCHL5 and USP14. NAC completely reverses auranofin-induced Ub-prs accumulation, caspase activation and PARP cleavage in HepG2 and MCF-7 cells which indicates that the proteasome inhibition is required for auranofin-induced cytotoxicity.

To preclude the ROS generation-induced cell death, we use Tbhq, a phenol-containing antioxidant that cannot bind the active atom site of auranofin, to scavenge auranofin-mediated ROS generation while not blocking auranofin-mediated proteasome inhibition and PARP cleavage. Auranofin also interferes with multiple proteasome-related signal proteins such as CHOP, caspase 12, IκB-α and NF-κB p65. Daily treatment of mice bearing HepG2 and MCF-7 xenografts with 6 mg/kg/day auranofin for 15 and 21 days resulted in significant tumor growth inhibition. Associated with this growth inhibition, a significant accumulation of ubiquitinated proteins, Ub-prs, K48/K63-linked proteins, c-Jun, p21 are observed that shows the auranofin-mediated tumor growth and proteasome function inhibition. Moreover, auranofin can selectively induce cytotoxicity in primary monocytes from patients with AML (average 24h IC50=0.159 μM while CTR=0.622 μM). Furthermore, our recent study reveals that auranofin overcomes Imatinib mesylate resistance through both Bcr/Abl-dependent and -independent mechanisms, and proteasome inhibition but not ROS is required for auranofin-induced caspase activation and apoptosis [99].

Therefore, CuPT can potently inhibit the 19S proteasome-associated UCHL5 and USP14 DUB activities at 0.5 μM dose, while it can also directly inhibit 20S proteasome activities at relatively higher doses than their cytotoxic doses in the cell. The latter inhibition might be related to the direct copper binding to proteasome peptidase subunits in the cell. However, under our experimental conditions, auranofin does not inhibit the activities of chymotrypsin-like, trypsin-like or caspase-like activities of 20S proteasomes which is different from the 20S proteasome inhibitors such as bortezomib. We suggest that Auranofin is the first DUB inhibitor reported to treat human disease in clinical use. We are currently using various complexes of metals including cadmium, zinc, nickel and platinum containing the same chelating ligand pyrithione to compare their effects on the UPS. The results of these experiments might be able to lead to a discovery of the clinical potential metal-based proteasome inhibitors for cancer therapy.

## CONCLUSIONS AND OUTLOOKS

In summary, we suggest the applications of the copper/gold complexes as a class inhibitors of deubiquitinates including the 19S proteasome-associated deubiquitinases. We have demonstrated that CuPT and auranofin are the novel metal inhibitors of UCHL5 and USP14 of the 19S proteasome by using several tumor models, including hematological malignancies, several solid tumor models and primary cells of the acute myeloid leukemia patients. We expect that the metal complexes could be developed to the potent inhibitors of 19S proteasome-associated deubiquitinases and be used for treating cancer and many other human diseases.
